# The N-terminal dimerization is required for TDP-43 splicing activity

**DOI:** 10.1038/s41598-017-06263-3

**Published:** 2017-07-21

**Authors:** Lei-Lei Jiang, Wei Xue, Jun-Ye Hong, Jun-Ting Zhang, Min-Jun Li, Shao-Ning Yu, Jian-Hua He, Hong-Yu Hu

**Affiliations:** 1 State Key Laboratory of Molecular Biology, CAS Center for Excellence in Molecular Cell Science, Shanghai Institute of Biochemistry and Cell Biology, Chinese Academy of Sciences; University of Chinese Academy of Sciences, 320 Yueyang Road, Shanghai, 200031 P.R. China; 2 Department of Chemistry, Fudan University, 220 Handan Road, Shanghai, 200433 P.R. China; 3 Shanghai Institute of Applied Physics, Chinese Academy of Sciences, 239 Zhangheng Road, Shanghai, 201204 P.R. China

## Abstract

TDP-43 is a nuclear factor that functions in promoting pre-mRNA splicing. Deletion of the N-terminal domain (NTD) and nuclear localization signal (NLS) (i.e., TDP-35) results in mislocalization to cytoplasm and formation of inclusions. However, how the NTD functions in TDP-43 activity and proteinopathy remains largely unknown. Here, we studied the structure and function of the NTD in inclusion formation and pre-mRNA splicing of TDP-43 by using biochemical and biophysical approaches. We found that TDP-43 NTD forms a homodimer in solution in a concentration-dependent manner, and formation of intermolecular disulfide results in further tetramerization. Based on the NMR structure of TDP-43 NTD, the dimerization interface centered on Leu71 and Val72 around the β7-strand was defined by mutagenesis and size-exclusion chromatography. Cell experiments revealed that the N-terminal dimerization plays roles in protecting TDP-43 against formation of cytoplasmic inclusions and enhancing pre-mRNA splicing activity of TDP-43 in nucleus. This study may provide mechanistic insights into the physiological function of TDP-43 and its related proteinopathies.

## Introduction

**Figure 1:**
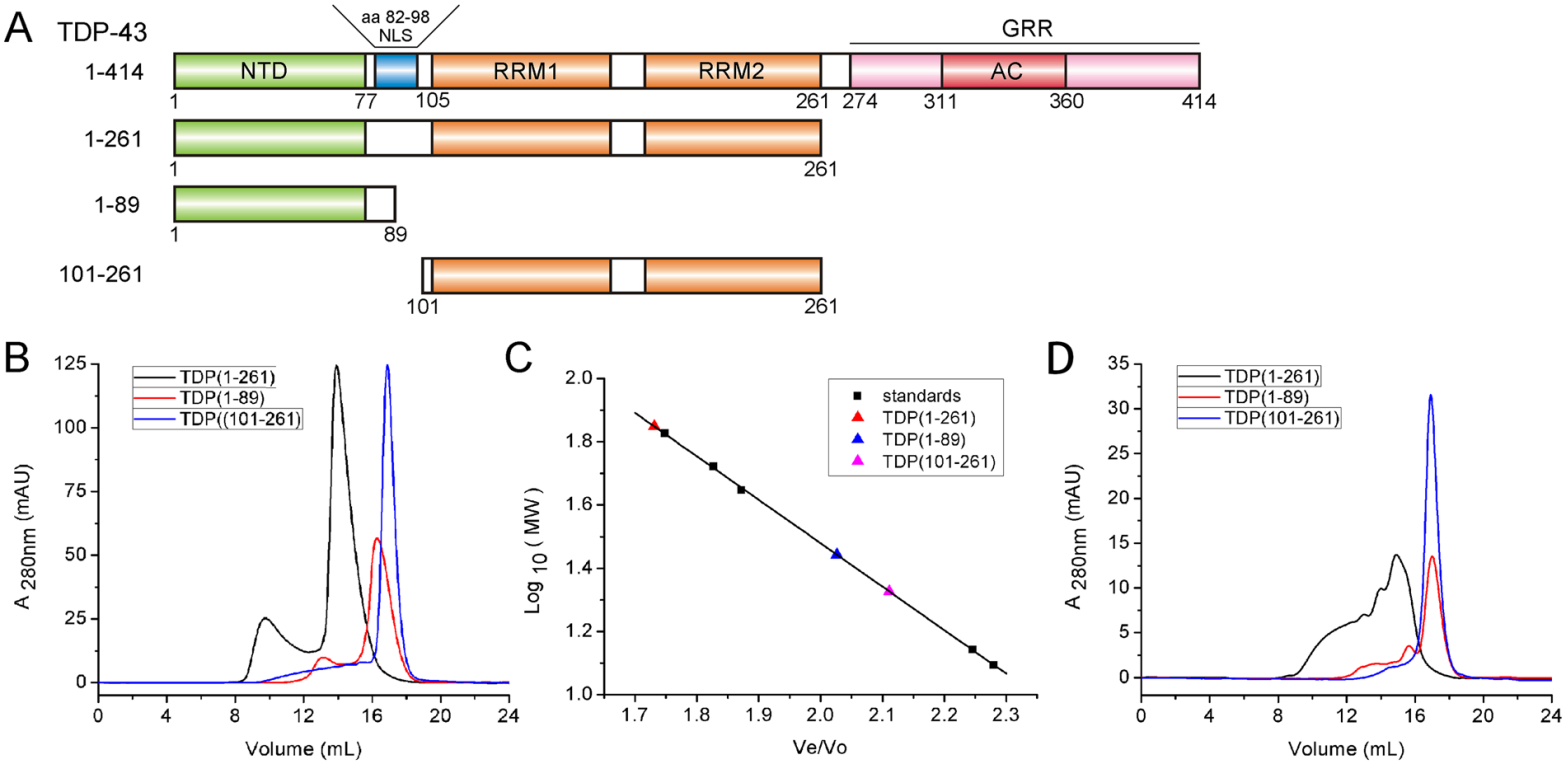
Characterization of the oligomeric states of the N-terminal fragments of TDP-43 by SEC. (**A**) Schematic representation of the domain architecture of TDP-43 and its N-terminal fragments. NTD, N-terminal domain; NLS, nuclear localization signal; RRM, RNA recognition motif; GRR, glycine-rich region; AC, amyloidogenic core. (**B**) SEC analysis of the N-terminal fragments of TDP-43. The proteins were diluted with Buffer A (25 mM Tris-HCl, pH 8.0, and 150 mM NaCl) to a concentration of 50 µM and loaded onto a Superdex-200 Increase 10/30 GL column. The chromatographic profiles are shown for TDP(1–261) (black), TDP(1–89) (red), and TDP(101–261) (blue). A_280nm_, Absorbance at 280 nm; mAU, micro absorbance unit. (**C**) Standard profile for calculating the apparent molecular weight (MW). V_e_/V_0_, relative elution volume. The standard markers are: BSA (67.0 kDa), GST (52.6 kDa), ovobumin (44.3 kDa), thioredoxin (13.9 kDa), and cytochrome C (12.4 kDa). The concentrations of the standards are around 10 to 100 μM. (**D**) As in (B), SEC profiles of the N-terminal fragments of TDP-43 at 10-µM concentration.

TDP-43 (TAR DNA-binding protein of 43 kDa) is a DNA/RNA binding protein belonging to the heterogeneous nuclear ribonucleoprotein (hnRNP) family. It was originally identified as a transcriptional repressor of the HIV-1 gene^1^. TDP-43 is highly conserved from human to *Caenorhabditis elegans* ^2^ and plays multiple roles in transcription, pre-mRNA splicing and processing, regulation of translation, and RNA stability^3–6^. From its primary structure, TDP-43 is composed of an N-terminal domain (NTD), two RNA recognition motifs (RRMs), and a long C-terminal glycine-rich region (GRR) (see Fig. 1A). The RRM domains are responsible for binding to UG and TG repeats of RNA and DNA^2, 7^, whereas the GRR mediates protein-protein interactions between TDP-43 and other hnRNP members^8, 9^. TDP-43 also contains a nuclear localization signal (NLS) and a nuclear export signal (NES), shuttling TDP-43 between the nucleus and the cytoplasm^10, 11^.

So far, TDP-43 has been shown to be the major component of inclusions associated with neurodegenerative diseases, including amyotrophic lateral sclerosis (ALS) and frontotemporal lobar degeneration (FTLD)^12, 13^. The disease-specific biochemical signatures of TDP-43 have been studied worldwide^10, 12, 13^. Under physiological conditions, TDP-43 localizes primarily in nucleus. However, it undergoes ubiquitination, redistribution from the nucleus to cytoplasm, N-terminal truncation and formation of cytoplasmic inclusions, and consequently loses its normal function in pathological cases, known as TDP-43 proteinopathies^10, 12–15^. The pathological mechanism induced by TDP-43 has been reviewed broadly^16–19^. In disease pathologies, the C-terminal fragments have been investigated considerably; they are essential for TDP-43 aggregation and inclusion formation^20–26^. Some studies have defined the Gln/Asn-rich domain^27, 28^ or amyloidogenic cores^29–31^ in the C-terminal region for TDP-43 aggregation. In addition, numerous mutations in TDP-43, mainly in the C-terminal region, have been identified in familial and sporadic cases of ALS and FTLD, and demonstrated to influence the aggregation and cellular function of TDP-43^14, 32–36^ and to alter axonal transportation^37, 38^.

Several studies have suggested that TDP-43 mainly forms a functional homodimer under physiological conditions, but it may transform to oligomers or aggregates, which lead to loss of function in pathological diseases^39–43^. To date, accumulating evidence has directed the research focusing on the importance of the C-terminal part for TDP-43 aggregation, whereas the biological and pathological roles of the N-terminus have been studied to a limited extent. In the past few years, some studies have revealed that the structure and structural integrity of the NTD are essential for its self-interaction and biological function^44–46^. Very recently, the solution structures of TDP-43 NTD have been elucidated by NMR under extreme conditions^47, 48^ and thoroughly compared and evaluated^49^. They are both canonical β-barrel structures constructed by seven β-strands and a short α-helix. In this regard, mechanistic study based on structural analysis of the NTD will be beneficial to understanding the biological roles played by TDP-43 under physiological and pathological conditions. In this communication, we further investigated the biochemical properties and physiological function of the NTD of TDP-43. We found that TDP-43 NTD forms a dimer or tetramer under normal conditions and inhibits TDP-43 aggregation. Disruption of dimerization can result in loss of TDP-43 splicing activity. This study may provide further insights into the biological function of the N-terminal dimerization in integration of TDP-43 conformation and its splicing activity, which is implicated in the pathological roles in neurodegenerative diseases.

## Results

### The N-terminal domain of TDP-43 forms a homodimer

It was proposed that TDP-43 forms a dimeric structure under physiological conditions and its NTD is capable of oligomerization and self-interaction^42–48^. Our previous study revealed that staurosporine (STS)-triggered removal of the N-terminus (including NTD and NLS) from TDP-43 (i.e., TDP-35) causes cytoplasmic localization and inclusion formation^20^. To obtain further insight into the physiological properties of TDP-43 NTD, we constructed different N-terminal fragments of TDP-43 (Fig. 1A) and performed size-exclusion chromatography (SEC) experiments (Fig. 1). As a result, the retention volume of the main peak of TDP(1–261) at a protein concentration of 50 µM was 13.92 mL (Fig. 1B), corresponding to an apparent molecular weight (MW) of 68.6 kDa as estimated based on the standard proteins (Fig. 1C). This apparent MW was close to the theoretical value for a dimer (2 × 30.7 kDa) (Table 1). The retention volume of the main peak of TDP(1–89) at the same concentration was 16.27 mL, corresponding to an MW of 27.1 kDa, also estimated to be a dimer. However, the apparent MW of TDP(101–261) was close to the theoretical value, suggesting that it forms a monomer in solution (Table 1). Considering the normal concentration of TDP-43 in cell and the limitation of detecting low-concentration proteins in SEC experiment, we determined the retention volume of the TDP-43 fragments at a 10-µM concentration. The three TDP-43 fragments exhibited elution profiles similar to the respective ones at the 50-µM concentration (Fig. 1D). We also observed the retention peaks of TDP(1–261) at different concentrations (Fig. S1A). With the increase of protein concentration, the retention peak gradually shifted to that of a larger MW, suggesting that TDP(1–261) altered its state from dimer to tetramer at a higher concentration. TDP(1–89) had a similar SEC profile to TDP(1–261) (Fig. S1B). However, TDP(101–261) exhibited almost the same retention volumes at different concentrations (Fig. S1C), suggesting that it forms a monomer in solution. Thus, the results from SEC experiments demonstrate that TDP-43 NTD is the source of dimerization and even further tetramerization of TDP-43.

**Table 1:** Apparent MWs of the N-terminal fragments of TDP-43 as determined by SEC.

Protein	Conc. (μM)	Volume (mL)	Apparent MW (kDa)	Theoretical MW (kDa)	n
TDP(1–261)	50	13.92	68.6	30.7	2.2
TDP(1–89)	50	16.27	27.1	11.1	2.4
TDP(101–261)	50	16.89	21.2	19.6	1.1

The Superdex-200 Increase 10/30 GL column was applied to SEC experiments. n, ratio of the apparent MW to theoretical MW; Conc., concentration.

### Formation of intermolecular disulfide promotes further tetramerization of TDP-43 NTD

In the SEC experiments, we also observed an additional minor peak corresponding to a larger MW oligomer eluted out before the major peak in the chromatograms for TDP(1–261) (Fig. S2A) and TDP(1–89) (Fig. S2B). However, when treated with 5 mM DTT, the minor peaks disappeared, suggesting that these large MW oligomers formed through intermolecular disulfides. On the contrary, DTT treatment had no effect on the monomeric state of TDP(101–261) (Fig. S2C). Therefore, we presumed that the Cys residues in TDP-43 NTD contribute to the oligomerization of TDP-43. To exclude the influence of disulfide formation on the oligomerization of TDP-43 NTD, we mutated both Cys39 and Cys50 to Ser residues. We observed that the additional peaks disappeared, as in the case of DTT treatment (Fig. S2D,E). Similar to that of the wild type, the main peaks of TDP(1–261) and TDP(1–89) mutants also had small shifts with increasing concentrations, but they still remained as dimers. Collectively, formation of the intermolecular disulfides by Cys residues is beneficial for tetramerization of TDP-43 NTD.

### TDP(1–77) is a stable dimer in solution

**Figure 2:**
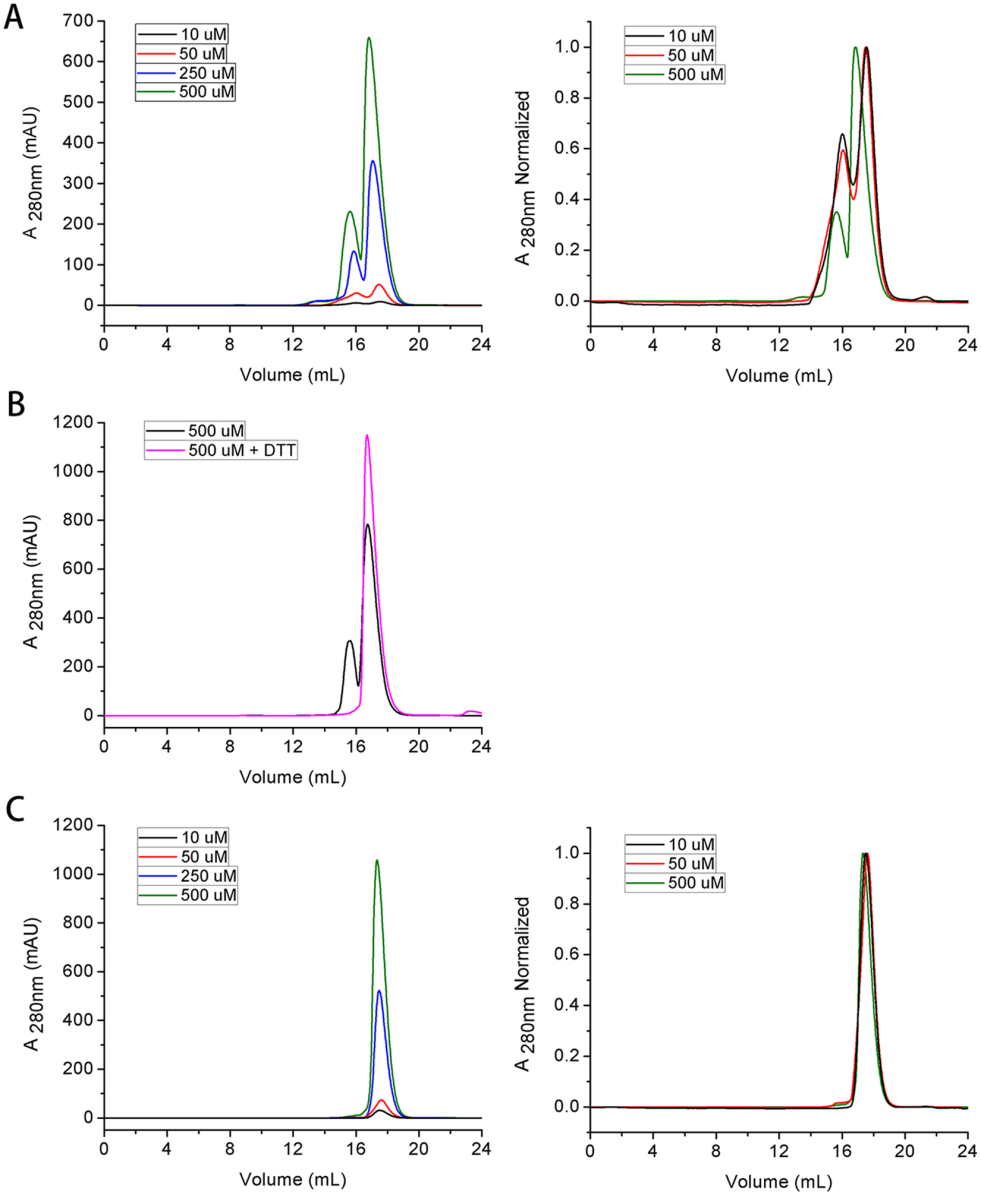
SEC profiles for TDP(1–77) and its Cys mutant. (**A**) SEC profiles for TDP(1–77) at different concentrations. (**B**) SEC profiles for TDP(1–77) with (pink) or without 5 mM DTT (black). The protein concentration was to 500 µM. (**C**) SEC profiles for TDP(1–77)-C39/C50S at different concentrations. The SEC experiments were carried out using a Superdex-200 Increase 10/30 GL column previously equilibrated with Buffer A. The right panels show the normalized chromatograms.

Because TDP(1–89) is not stable in solution, we prepared the N-terminal 77-residue construct (TDP(1–77)) for further structural analysis. With the increase of protein concentration, the SEC profile of TDP(1–77) had two peaks, a major and a minor peak (Fig. 2A). We estimated that the major peaks reflect the dimeric states, whereas the minor peaks correspond to the tetrameric states (Table 2). Moreover, the minor peak vanished when the sample was treated with 5 mM DTT (Fig. 2B). Interestingly, a Cys mutant (C39/C50S) of TDP(1–77) exhibited only one sharp peak at any concentration tested (Fig. 2C). The retention volume of the peak was ~17.6 mL corresponding to an MW of 16.0 kDa, suggesting that only the dimeric state existed in the Cys mutant.

**Table 2:** Apparent MWs of TDP(1–77) and its Cys mutants at different concentrations as determined by SEC.

Protein	Conc. (μM)	Volume (mL) major (minor)	Apparent MW (kDa)	Theoretical MW (kDa)	n
TDP(1–77)	10	17.55 (16.01)*	16.3 (30.0)	9.6	1.7(3.1)
	50	17.47 (16.04)	16.9 (29.7)	9.6	1.8(3.1)
	250	17.07 (15.88)	19.7 (31.6)	9.6	2.1(3.3)
	500	16.83 (15.64)	21.7 (34.8)	9.6	2.3(3.6)
	500 + DTT	16.71	22.8	9.6	2.4
TDP(1–77)-C39/C50S	10	17.50	16.7	9.6	1.7
	50	17.61	16.0	9.6	1.7
	250	17.47	16.9	9.6	1.8
	500	17.34	17.7	9.6	1.8

The Superdex-200 Increase 10/30 GL column was applied to SEC experiments. *Data in parentheses for minor peaks; n, ratio of the apparent MW to theoretical MW; Conc., concentration.

We next performed dynamic light scattering (DLS) analysis on TDP(1–77) and its Cys mutant to verify their size and size distribution (Fig. S3). TDP(1–77) gave a wide size distribution of roughly spherical particles with an average hydrodynamic radius of 3.1 nm and an apparent MW of ~47 kDa, suggesting that wild-type TDP(1–77) formed a large population of tetramers in solution. This is consistent with the previous observation from the SEC experiments (Table 2) that the particle size was gradually enlarged with the increase of protein concentration. However, the size distribution of TDP (1–77)-C39/C50S exhibited a single narrow peak in the DLS intensity profile. The average hydrodynamic radius was 2.3 nm and the apparent MW was ~21 kDa, strongly supporting formation of a stable dimer of the Cys mutant.

We also carried out non-reducing SDS-PAGE analysis to examine the effect of the two Cys residues on the oligomeric state of TDP-43 NTD. TDP(1–77) and its two single-Cys mutants (C39S, C50S) presented a band corresponding to the dimeric state in the absence of DTT, whereas the double mutant (C39/C50S) exhibited no band for dimeric state whether DTT was present or not (Fig. S4A). Based on the band intensity, Cys50 may be more accessible and prone to intermolecular disulfide formation than Cys39. This suggests that intermolecular disulfide formation contributes to dimerization or tetramerization. Circular dichroism (CD) spectroscopy indicated that disrupting the disulfide-induced oligomerization had little effect on the secondary structures of TDP(1–77) (Fig. S4B), although all TDP(1–77) variants gave the non-canonical CD spectra. Taken together, these biochemical results confirm that TDP-43 NTD is able to form dimers itself and further tetramers through disulfide formation, which is sufficient for leading full-length TDP-43 to dimerization and tetramerization.

### Solution structure of TDP-43 NTD

**Figure 3:**
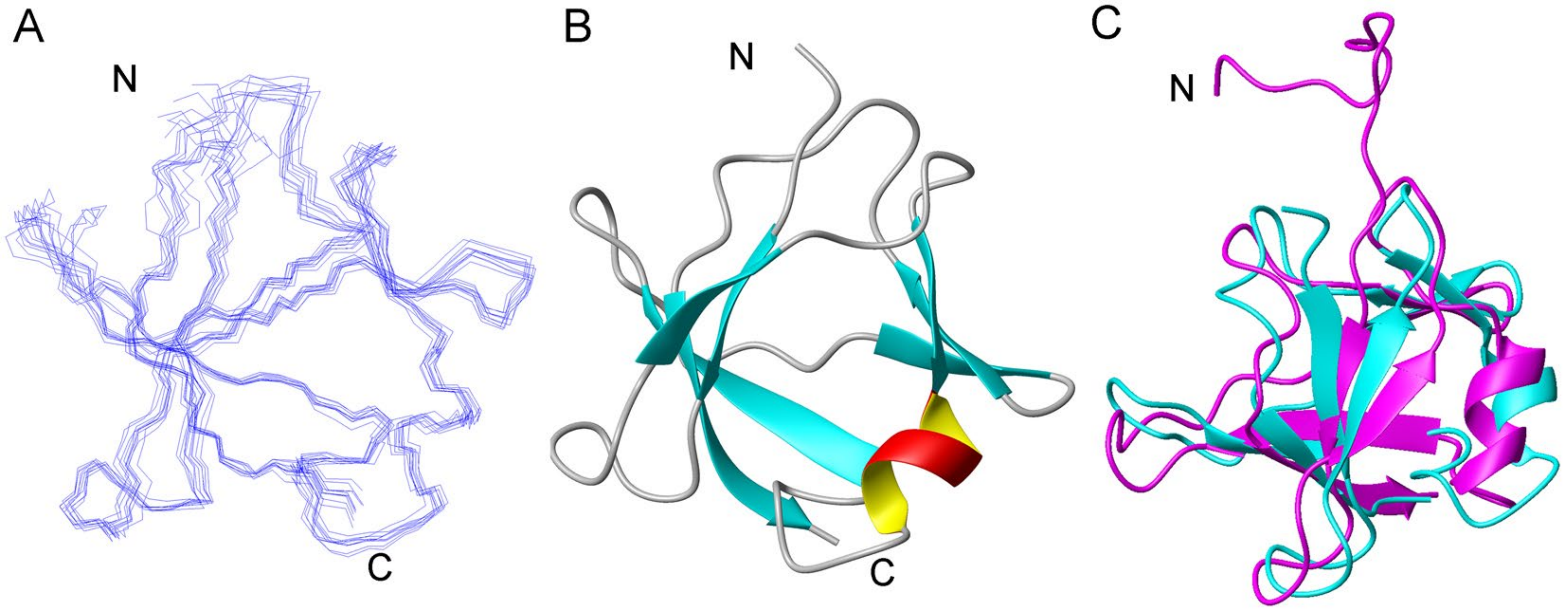
Solution structure of TDP(1–77) (C39/C50S) elucidated by NMR. (**A**) Superposition of the backbone traces of the 10 lowest-energy structures. (**B**) Ribbon diagram of a representative structure of TDP(1–77) (C39/C50S), showing seven β-strands and one short α-helix. The structure was elucidated with the GB1-fused Cys mutant TDP(1–77)-GB1-C39/C50S. (**C**) Comparison of the overall structures of TDP(1–77) from TDP(1–77)-GB1-C39/C50S (blue) and His6-TDP(1–77) (pink; PDB: 2N4P). The RMSD is 2.96 Å. N, N-terminus; C, C-terminus. All the structures are displayed using MOLMOL.

To gain further insights into the dimerization of TDP-43 NTD, we solved its structure in solution by using NMR techniques. In order to improve its stability and homogeneity for NMR data acquisition, we fused GB1 to the C-terminus of TDP(1–77) (TDP(1–77)-GB1). Meanwhile, we also applied SEC, DLS and non-reducing SDS-PAGE to confirm the stability and homogeneity of TDP(1–77)-GB1 and its Cys mutant (Fig. S5). TDP(1–77)-GB1 exhibited similar biochemical characteristics to TDP(1–77), supporting the existence of dimers with a small population of tetramers in solution (Fig. S5A,B,D). As the Cys residues (C39, C50) contribute to the formation of tetramer from dimer, which may influence the homogeneity of the protein, we applied a Cys mutant (TDP(1–77)-GB1-C39/C50S) to structural analysis by NMR. As expected, TDP(1–77)-GB1-C39/C50S was still in the stable dimeric state under an NMR condition (Fig. S5C,E). We assigned the chemical shifts and unambiguous intramolecular NOEs to calculate the monomeric structure. The restraints used for structure calculation and statistics are summarized in Table S1. In general, TDP(1–77) forms a β-barrel structure mainly comprised of two β-sheets (β1β2β4β7, β3β5β6) with a short α-helix between them (Fig. 3A,B). Overall, it shows structural fold and secondary-structure pattern similar to the previously reported structure (PDB ID: 2N4P) solved in dilute protein concentration^48^, giving an RMSD of 2.96 Å. Besides some difference in the flexible regions, the α-helix and β3-strand are two-residue shorter than those of 2N4P, whereas the β6-strand is two-residue longer. However, this structure is largely different from that solved under the pure-water condition^47^.

### The dimeric interface of TDP-43 NTD

**Figure 4:**
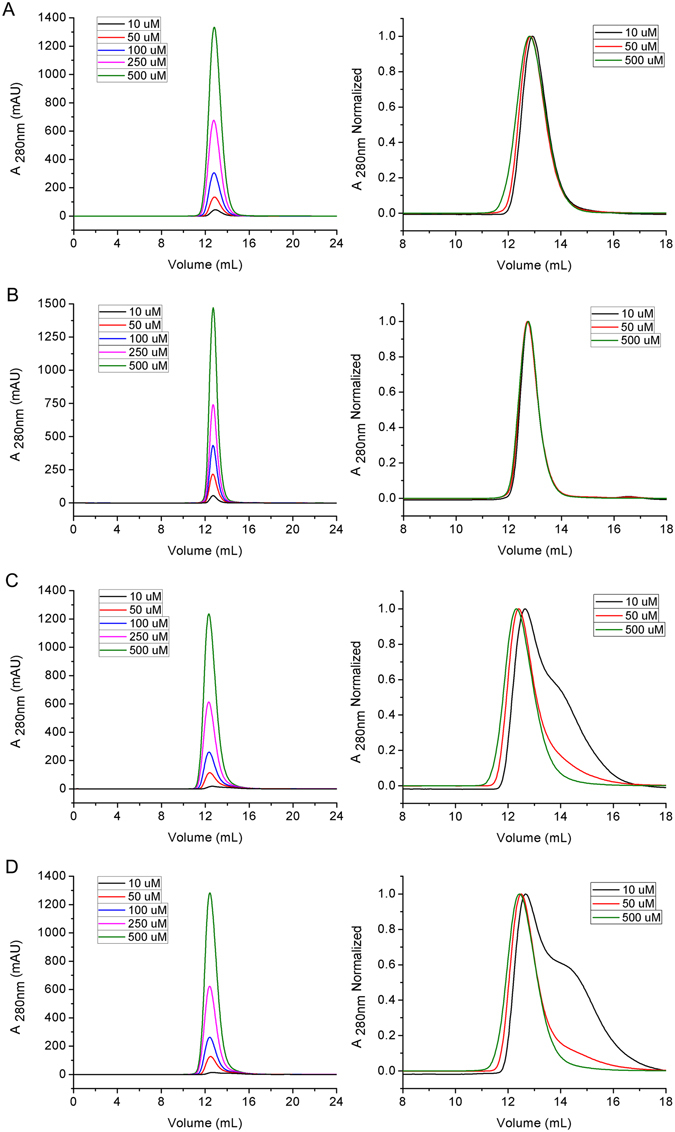
Identification of the dimer interfaces of TDP(1–77) by mutagenesis and SEC. (**A**) SEC profiles for TDP(1–77)-GB1-C39/C50S. (**B**) L27/I60/V57A mutant. (**C**) L71/Y73A mutant. (**D**) V72/V74/Y43A mutant. The right panels show the normalized chromatograms. The mutants were generated based on TDP(1–77)-GB1-C39/C50S. The proteins were diluted to the indicated concentrations with Buffer A and loaded onto a Superdex-75 10/30 GL column.

TDP-43 NTD forms predominantly dimeric structures under normal conditions, but the residues that are crucial for dimerization are unknown. To address this issue, we prepared several mutants based on the monomeric structure (Fig. 3) and the predicted interfaces of TDP(1–77), and performed SEC analysis (Fig. 4). As a result, TDP(1–77)-GB1-C39/C50S eluted with a single peak corresponding to an apparent MW of 25.6 kDa (Fig. 4A), suggesting it formed a folded dimer. The L27A/I60A/V57A mutant (mutation around the α-helix) was well in line with TDP(1–77)-GB1-C39/C50S (Fig. 4B), also eluted as an apparent dimer. However, additional peaks corresponding to monomers eluted after the main peaks both for L71A/Y73A and V72A/V74A/Y43A mutants (mutation mainly in the β7 strand) at low protein concentrations, implying that these two mutants had their dimers partly dissociated to monomers (Fig. 4C,D). These results suggest that the residues of Leu71-Val74 in the β7-strand are mainly clustered in the dimer interface, and they are crucial for TDP-43 NTD dimerization.

Because CD spectroscopy could not provide secondary structure information for TDP (1–77) (Fig. S4B), we resorted to Fourier transform infrared (FT-IR) spectroscopy to compare the secondary structures of TDP (1–77) and its mutants. Quantitative analysis revealed that the β-sheet contents of the L71/Y73A and V72/V74/Y43A mutants were roughly unchanged, as compared with those of TDP(1–77) and its C39/C50S mutant (Fig. S6). Moreover, the secondary structure content of TDP(1–77)-C39/C50S estimated from FT-IR was consistent with that calculated from our NMR structure (PDB ID: 5X4F). These results suggest that transformation of the oligomeric states, tetramer to dimer to monomer, has no considerable influence on the secondary structures of TDP-43 NTD.

### The N-terminal dimerization inhibits formation of TDP-43 inclusions in cytoplasm

**Figure 5:**
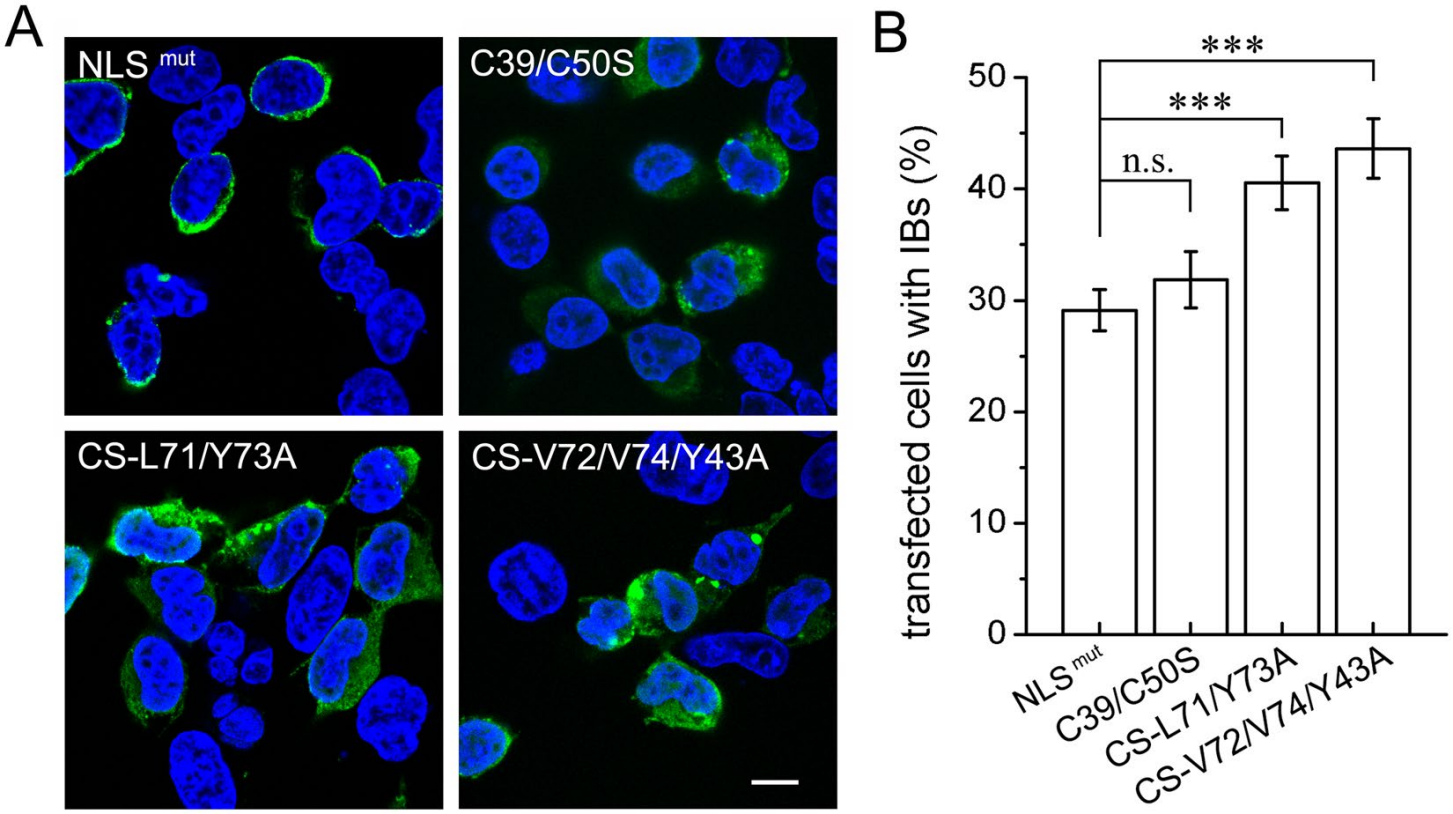
Immunofluorescence microscopic imaging of cytoplasmic inclusions of TDP-43-NLS^mut^ and its dimer-interface mutants. (**A**) Inclusion formation of FLAG-TDP-43-NLS^mut^ and its Cys mutant (C39/C50S) and dimer-interface mutants (CS-L71/Y73A and CS-V72/V74/Y43A) in HEK 293 T cells. TDP-43-NLS^mut^ and its mutants were stained with mouse anti-FLAG (green), and the nuclei were stained with Hoechst (blue). The images are shown with a magnification of 3 times (3 X). Scale bar = 10 µm. The mutants were generated based on FLAG-TDP-43-NLS^mut^. (**B**) Quantification of the cells with cytoplasmic inclusions in HEK 293 T cells overexpressing FLAG-TDP-43-NLS^mut^ or its mutants. Cells were counted and the data were statistically analyzed by one-way ANOVA. Results are presented as Mean ± SEM (n = 25–30). ***p < 0.001; n.s., no significance.

Our previous study revealed that TDP-35 mislocalizes in the cytoplasm and forms inclusions due to removal of the NLS and NTD. To further obtain information of TDP-43 NTD dimerization in cytoplasmic inclusion formation, we prepared Cys-to-Ser and dimer-interface mutants based on the NLS mutant (NLS^mut^) construct of TDP-43 for confocal microscopy imaging (Fig. 5). As known, all the mutants were mainly localized in the cytoplasm, and the NLS^mut^ mutant formed inclusions in few cells (Fig. 5A). The Cys mutations (C39/C50S) had very little influence on inclusion formation, whereas the two dimer-interface mutants (L71/Y73A, V72/V74/Y43A) significantly enhanced the formation of cytoplasmic inclusions (Fig. 5A,B). These results indicate that the N-terminal dimerization is able to protect TDP-43 against formation of cytoplasmic inclusions.

### The N-terminal dimerization of TDP-43 plays a role in its splicing activity

**Figure 6:**
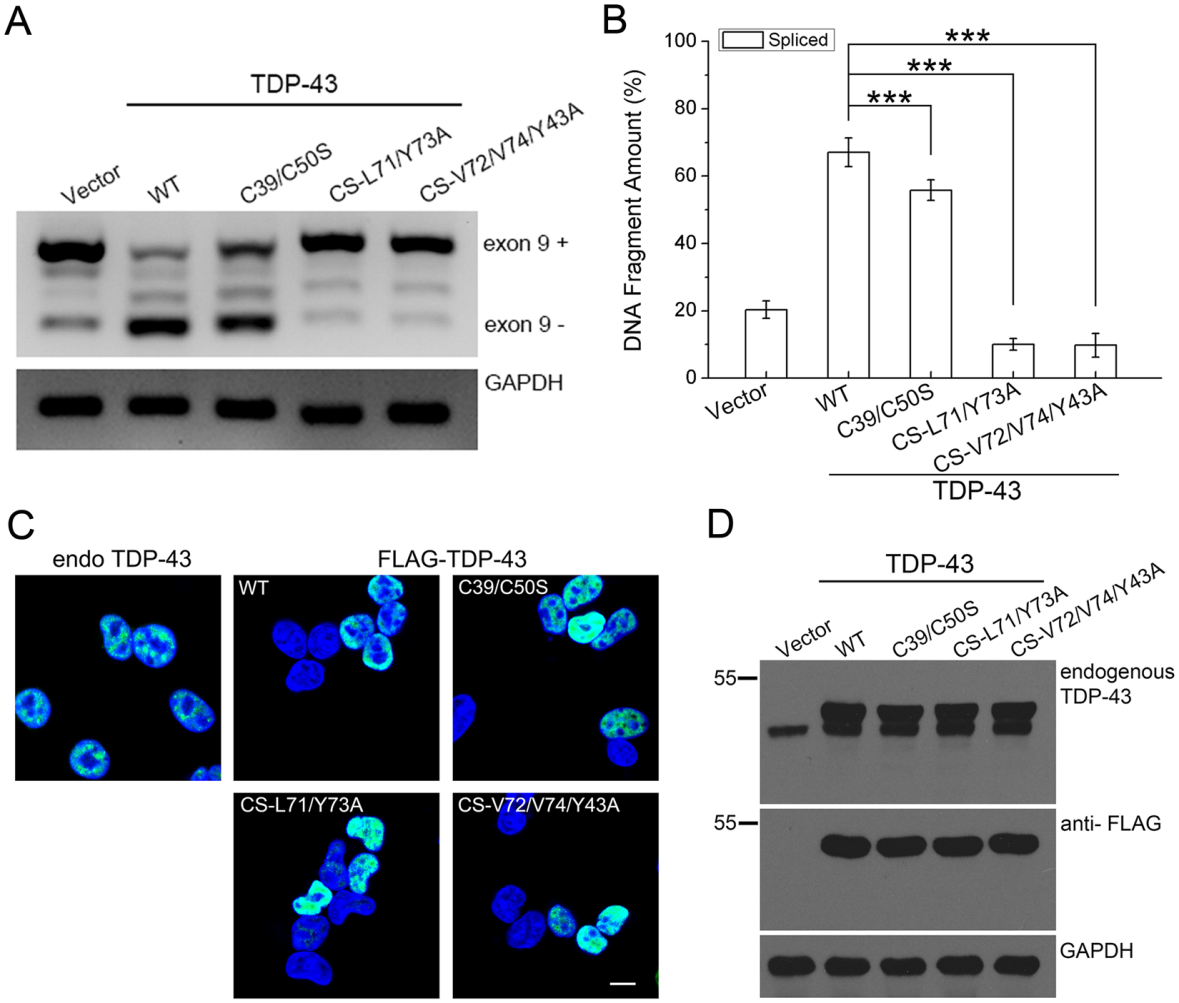
Effects of dimer-interface mutations on the RNA splicing activity of TDP-43. (**A**) Effects of TDP-43, its Cys mutant (C39/C50S) and dimer-interface mutants (CS-L71/Y73A and CS-V72/V74/Y43A) on CFTR exon 9 splicing. The GT13T5 reporter was co-transfected with the FLAG-tagged plasmids into HEK 293 T cells, respectively. Exon 9 + stands for unspliced cDNA fragment, while exon 9− denotes spliced cDNA. (**B**) Quantification of the spliced RT-PCR products of TDP-43 and its mutants based on band intensity. Data were analyzed by one-way ANOVA and represented as Mean ± SD (n = 3). ***p < 0.001. (**C**) Immunofluorescence microscopic images showing nuclear localization of FLAG-TDP-43 and its mutants in HEK 293 T cells. TDP-43 and its mutants were stained with mouse anti-TDP-43 or anti-FLAG (green), and the nuclei were stained with Hoechst (blue). The images are shown with a magnification of 3 times (3 X). Scale bar = 10 µm. (**D**) Western blot showing the expression levels of the protein species for CFTR splicing assay.

A well-known function of TDP-43 is its ability to promote alternative splicing^7, 50^. To evaluate the role of NTD dimerization in the splicing activity of TDP-43, we performed a CFTR exon 9 splicing assay^20^. We co-transfected the CFTR mini-gene reporter and TDP-43 or its mutants into HEK 293 T cells and compared their splicing activities (Fig. 6). As expected, overexpression of wild-type TDP-43 markedly enhanced CFTR exon 9 exclusion. However, transfection of TDP-43-C39/C50S led to a decrease in CFTR exon 9 exclusion compared to wild-type TDP-43 (Fig. 6A,B) under the conditions of comparable nuclear localization (Fig. 6C) and similar expression levels (Fig. 6D). Furthermore, when the dimer interface was disrupted by mutation of key residues (L71/Y73A or V72/V74/Y43A), the CFTR exon 9 splicing activity was almost abolished, in contrast to the partial reduction of CFTR exon 9 skipping activity caused by C39/C50S mutation (Fig. 6A,B). These results suggest that the dimer-interface mutants lose their splicing activity due to impairment of the functional homodimer. Therefore, our findings demonstrate that the N-terminal dimerization of TDP-43 is critical for its biological function.

## Discussion

TDP-43 is a nuclear factor that functions in promoting pre-mRNA splicing^4^. The C-terminal part has been reported to have RNA binding ability and act as the source for aggregation. TDP-35, deletion of the N-terminal NTD and NLS, may experience mislocalization to cytoplasm and cause formation of inclusions^20^. The present study reveals that the N-terminal dimerization of TDP-43 is essential for its splicing activity. Zhang *et al*. previously reported that the extreme N-terminal 10 residues are important for TDP-43 function in the nucleus^46^. It is probably that deletion of the N-terminal 10 residues disrupts the structural ensemble of TDP-43 NTD and consequently result in functional loss. Moreover, GFP fusion may alter the aggregation properties of TDP-43.

TDP-43 NTD is a well folded domain, and its solution structure has been elucidated by NMR under extreme conditions^47, 48^. TDP(1–77) is not homogeneous in solution, so we used the GB1-fusion strategy to solve the structure^51^. Biochemical and biophysical studies showed that TDP(1–77) remains a stable dimer under the experimental conditions. Different from the structure solved in a dilute solution and presented as a monomeric state^48^, we selected the intramolecular NOEs to calculate the monomeric structure from a stable dimeric state, analyzed the dimer interface by mutagenesis and SEC, and constructed the dimer structure via computer modeling (Fig. S7). Nevertheless, the monomeric structure is overall similar to that from Laurents’ group (PDB ID: 2N4P), despite different protein samples and approaches being applied. Generally, TDP-43 NTD forms a β-barrel structure comprised mainly of two β-sheets connected by a short α-helix.

**Figure 7:**
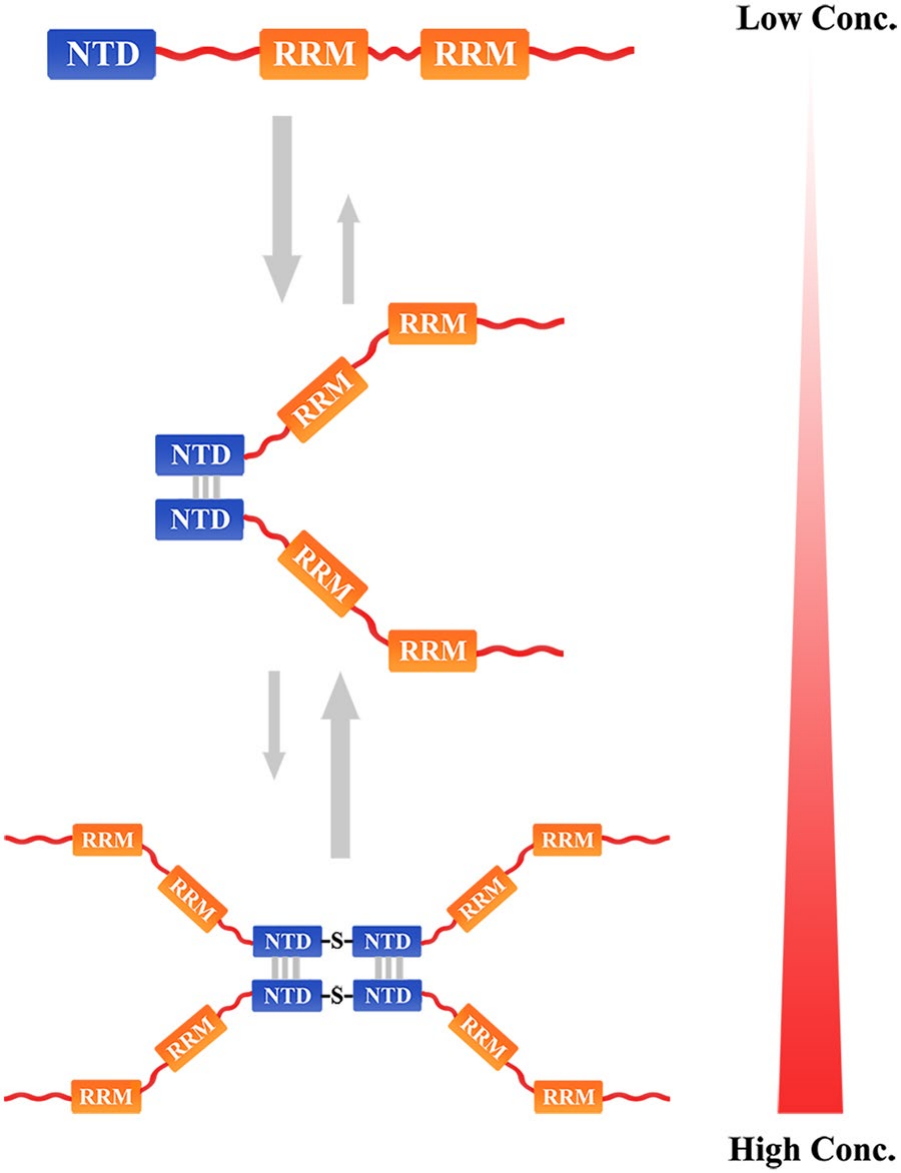
Schematic representation for the oligomerization of TDP-43. The model proposes that TDP-43 NTD mainly forms a dimer via residues Leu71 and Val72 around the β7-strand, and then transforms into a tetramer via disulfide formation at high protein concentrations. The NTD dimerization or tetramerization may protect TDP-43 against aggregation and is required for splicing activity of TDP-43.

We found that a hydrophobic interface resides around the β7-strand and plays a role in NTD dimerization. With increased protein concentration, TDP-43 transforms to a tetramer through disulfide formation (Fig. 7). Both the dimeric and tetrameric forms of full-length TDP-43 have splicing activity in nucleus (Fig. 6). The formation of dimers or tetramers seems to protect TDP-43 against aggregation and inclusion formation in cytoplasm (Fig. 5). Indeed, TDP-43 is a highly abundant protein localized in the nucleus^2, 10, 50^. Thus, we speculate that TDP-43 forms a dimer or tetramer under physiological conditions for pre-mRNA splicing in nucleus. However, TDP-35, a C-terminal fragment of TDP-43 without NTD and NLS, can induce its cytoplasmic localization, aggregation or inclusion formation, and further loss of RNA processing activity^20^. In addition, TDP-43 can also form nuclear or cytoplasmic granules under stress conditions, such as heat or arsenite treatment^52, 53^. Formation of the nuclear granules might have relevance to the nuclear splicing activity of TDP-43. However, whether the N-terminal dimerization of TDP-43 affects formation of nuclear stress granules remains future investigation.

The major finding of the present study is that the NTD fold of TDP-43 is essential for its dimerization and consequent splicing activity, whereas the monomeric or aggregated forms may lose their biological functions. This may provide mechanistic insights into the physiological function of TDP-43 and its related proteinopathies.

## Materials and Methods

### Construction of plasmids

The intrinsic *Nde* I site in the cDNA for human TDP-43 was previously mutated by nonsense mutation. The cDNAs of TDP-43 fragments and their mutants were digested and ligated into pET22b vector using *Nde I/Xho I* cloning sites. To prepare the GB1-fused C-terminal fragments, the cDNAs of TDP(1–77) and its mutants were subcloned into a pET22b-GB1 plasmid. For eukaryotic expression, TDP-43 and its mutants were constructed into pcDNA3.1/FLAG vector (Invitrogen) respectively. The Lys and Arg residues (K82, R83, K84, K95, K97 and R98) in the NLS sequence of TDP-43 were mutated to Ala for cytoplasmic expression. All the mutants were generated by site-directed mutagenesis and verified by DNA sequencing.

### Protein expression and purification

All proteins were overexpressed in *Escherichia coli* BL21 (DE3) strain (Invitrogen) and purified as described elsewhere^29, 32^. The His-tagged proteins were initially purified through a Ni^2+^-NTA column (Roche), followed by SEC (Superdex-75 or Superdex-200, GE Healthcare). The protein concentrations were determined spectrophotometrically using each extinction coefficient (ε_280_).

### Analytical size exclusion chromatography (SEC)

All SEC experiments were performed on the AKTA purifier facility with a fast protein liquid chromatography (FPLC) system (GE Healthcare) at room temperature. The target proteins were diluted to different indicated concentrations with Buffer A (25 mM Tris-HCl, pH 8.0, and 150 mM NaCl) and stored at 4 °C overnight. An aliquot of each sample was treated with 5 mM DTT. Before loading, all samples were centrifuged at 15,000 g at 4 °C for 5 min. Each 500-µL sample was applied to a Superdex-200 Increase 10/300 GL column or a Superdex-75 10/300 GL column (GE Healthcare) previously equilibrated with Buffer A, and eluted with the same buffer at a flow rate of 0.7 or 0.8 mL/min. Standard proteins were analyzed under the same conditions. The elution volume of each fraction in the profile was monitored by recording the absorbance at 280 nm.

### Dynamic light scattering (DLS)

The size and size distribution of proteins were determined by DLS on a DynaPro instrument (Wyatt Technology Co., Santa Barbara) in disposable 100-μL micro cuvettes. The proteins were diluted to different indicated concentrations with Buffer A. After centrifugation at 15,000 g at 4 °C for 30 min, the supernatants were subjected to DLS measurement. The scattering angle was fixed at 90° at a constant temperature of 25 °C. Data were collected for 30 scans and processed by Dynamics V7 software. The radius and size distribution were calculated using the regularization algorithm provided by the software.

### NMR spectroscopy and structure calculation

The C-terminal GB1 fusion was applied to improve the homogeneity of the protein studied^29, 51^. To solve the solution structure of TDP(1–77)-GB1-C39/C50S by NMR, the ^15^N/^13^C-labeled sample (~1 mM) was dissolved in Buffer B (20 mM phosphate, 50 mM NaCl, pH 6.5 and 8% D_2_O) for data acquisition. All NMR data were acquired on a Bruker Avance 600-MHz spectrometer equipped with a TCI CryoProbe (Bruker Biospin) at 25 °C. The chemical-shift and NOE assignments, data processing, and structure calculations were performed following the protocol for NMR structural analysis^29^.

### Fourier transform infrared spectroscopy (FT-IR)

FT-IR spectra were recorded on an ABB Bomem MB-3000 spectrometer (Quebec, Canada) equipped with a deuterated triglycine sulfate detector. Data processing was carried out as described elsewhere^54^. Second-derivative spectra were obtained using Savitsky-Golay derivative function software for a 7-data-point window^55^. The second-derivative amide I spectra were analyzed to estimate the relative secondary structure contents by manually computing the areas under the bands assigned to a particular substructure.

### Cell culture, transfection, Western blotting, and immunofluorescence microscopy

HEK 293 T cells were cultured in DMEM media (Invitrogen) supplemented with 10% fetal bovine serum (Gibco) and penicillin-streptomycin under an ambience of 5% CO_2_ at 37 °C. Transfection with plasmid was performed using PolyJet^TM^ reagent (SignaGen) according to the manufacturer’s instructions, and then the cells were harvested at 48 h after transfection. To examine the protein expression level, the transfected cells were lysed with a RIPA buffer (50 mM Tris-HCl, pH 7.5, 150 mM NaCl, 1 mM EDTA, 1% NP-40, and cocktail protease inhibitor (Roche Applied Science)) on ice for 30 min, and then centrifuged at 15,000 g at 4 °C for 15 min. The lysates were mixed with the 2x loading buffer and subjected to Western blotting. The antibodies included mouse monoclonal anti-FLAG (Sigma), mouse anti-TDP-43 (Abnova), mouse anti-GAPDH (Zen BioScience), and goat anti-mouse IgG-HRP (Jackson Immuno-Research). The proteins were detected by an ECL detection kit (Thermo scientific). For immunofluorescence microscopy, HEK 293 T cells were grown on glass cover slides for 48 h after transfection. The adherent cells were fixed with 4% paraformaldehyde for 15 min, permeabilized with 0.1% Triton X-100 for 1 h and blocked with 5% BSA/10% FBS for 1 h. The fixed cells were then incubated with an antibody against FLAG or TDP-43 for 1 h. After washing with PBS, the cells were labeled with an FITC-conjugated anti-mouse antibody (Jackson Immuno-Research) for 1 h, and the nuclei were stained with Hoechst 33258 (Sigma) simultaneously. The images were obtained using a Leica TCS SP4 confocal microscope (Leica Microsystems).

### CFTR exon 9 splicing assay

The splicing assay was performed on the CFTR mini-gene reporter TG13T5 as described previously^7, 20, 29, 50^. Briefly, the reporter plasmid was co-transfected with TDP-43 or its mutants into HEK 293 T cells. After 48 h, the cells were harvested and divided into two equal fractions. One was subjected to Western blotting to examine the expression level, and the other was used to measure the CFTR exon 9 splicing activity. The total RNA was extracted with TRIzol reagent (Invitrogen) and the cDNA was generated by retro-transcription using a ReverTra Ace-α kit (TOYOBO). PCR and agarose-gel electrophoresis were following our previous protocols^20^. The intensity of each band was quantified by Scion Image software (Scion Corp). The total intensity of exon 9+ and exon 9− of the vacant vector was set as a control, and the intensities of band exon 9− were compared with the control.

### Coordinates and﻿ structure factors

The coordinates and structure factors have been deposited in the Protein Data Bank with accession code 5X4F for TDP(1–77).

## Electronic supplementary material

Supplementary Information
