# Supplementary material for: The N-terminal dimerization is required for TDP-43 splicing activity
Source: Sci Rep. 2017 Jul 21;7:6196. doi: 10.1038/s41598-017-06263-3 (PMC5522446; doi:10.1038/s41598-017-06263-3)
Supplement: Supplementary file 1 — Supplementary Information [file 41598_2017_6263_MOESM1_ESM.pdf]

The N-terminal dimerization is required for TDP-43 splicing activity

Lei-Lei Jiang [1], Wei Xue [1], Jun-Ye Hong [1], Jun-Ting Zhang [2], Min-Jun Li [3], Shao-Ning Yu [2], Jian-Hua He [3], and Hong-Yu Hu [1]

[1] State Key Laboratory of Molecular Biology, CAS Center for Excellence in Molecular Cell Science, Shanghai Institute of Biochemistry and Cell Biology, Chinese Academy of Sciences; University of Chinese Academy of Sciences, 320 Yueyang Road, Shanghai 200031, P. R. China.

[2] Department of Chemistry, Fudan University, 220 Handan Road, Shanghai 200433, P. R. China.

[3] Shanghai Institute of Applied Physics, Chinese Academy of Sciences, 239 Zhangheng Road, Shanghai 201204, P. R. China.

Correspondence and requests for materials should be addressed to H.-Y H. (email: hyhu@sibcb.ac.cn).

SUBJECT AREAS: MECHANISMS OF DISEASES; PROTEIN AGGREAGATION

Running title: Structure and function of TDP-43 N-terminus

Figure S1

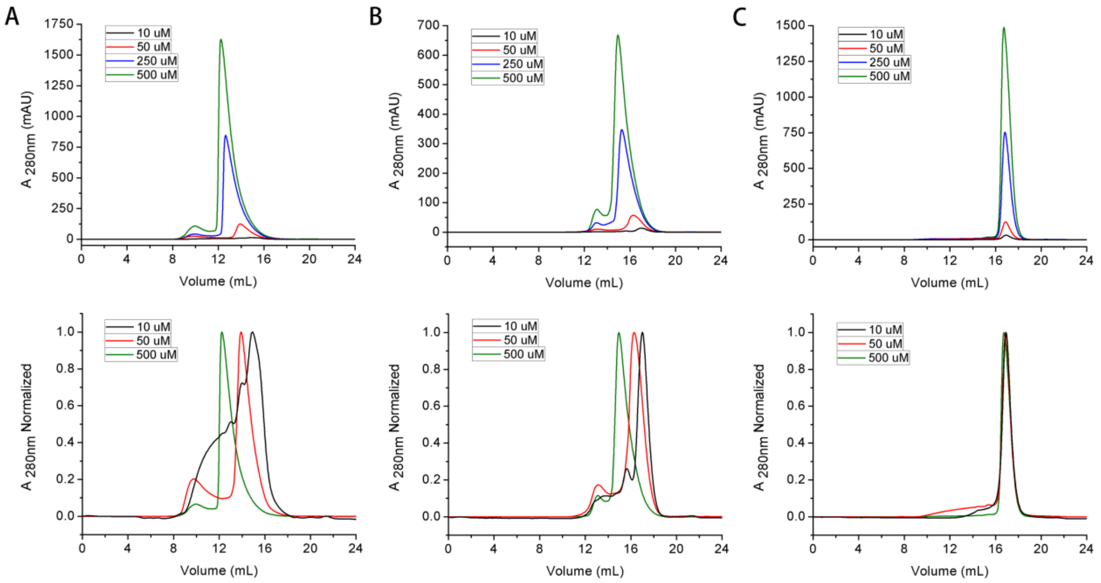

D

Protein	Conc. (μM)	Volume (mL)	Apparent MW (kDa)	Theoretical MW (kDa)	n
TDP(1-261)	10	14.90	46.5	30.7	1.5
	50	13.92	68.6	30.7	2.2
	250	12.64	113.6	30.7	3.7
	500	12.24	133.2	30.7	4.3
TDP(1-89)	10	17.00	20.3	11.1	1.8
	50	16.27	27.1	11.1	2.4
	250	15.29	39.8	11.1	3.6
	500	14.94	45.7	11.1	4.1
TDP(101-261)	10	16.91	21.0	19.6	1.1
	50	16.89	21.2	19.6	1.1
	250	16.82	21.7	19.6	1.1
	500	16.73	22.5	19.6	1.1

Figure S1. SEC characterization of the oligomeric states of the N-terminal fragments from TDP-43. (A) SEC profiles for TDP(1-261) at different concentrations. (B) TDP(1-89). (C) TDP(101-261). The down panels show the normalized chromatograms. The proteins were diluted to different concentrations with Buffer A and analyzed using a Superdex-200 Increase 10/30 GL column. (D) Data from the SEC experiments (A - C) on the apparent MWs and their stoichiometries (n). Conc., concentration.

Figure S2

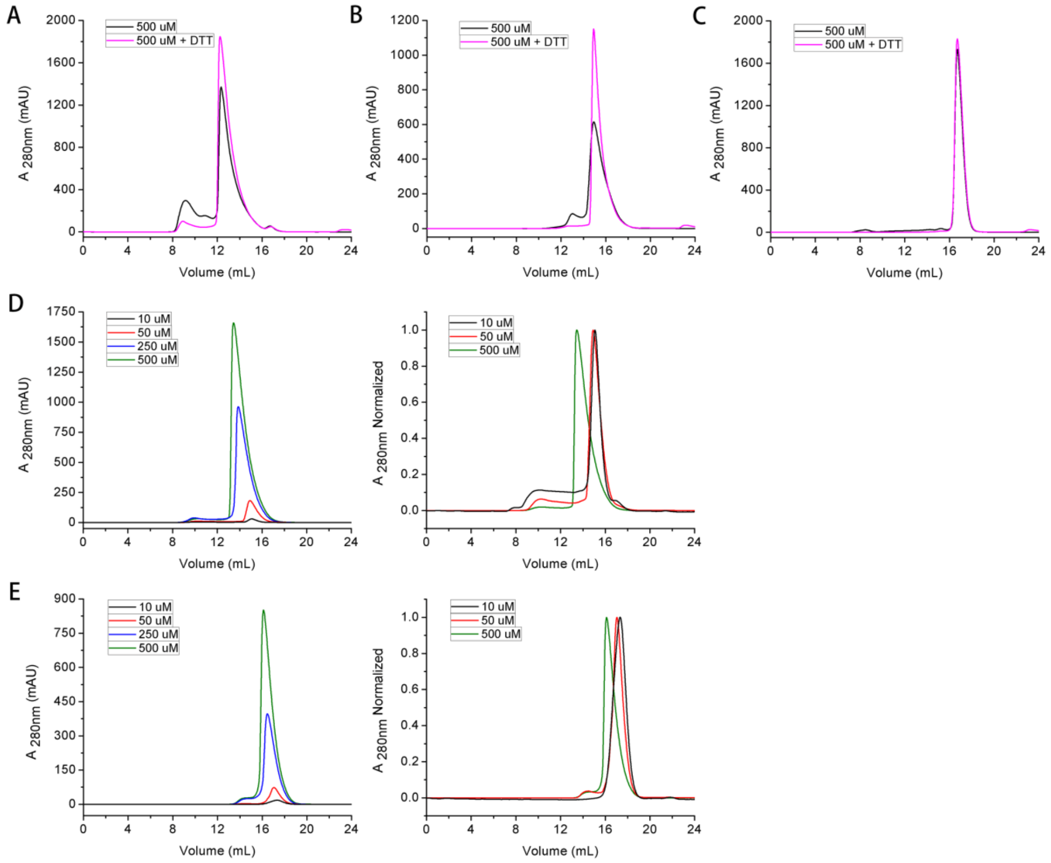

F

Protein	Conc. (μM)	Volume (mL)	Apparent MW (kDa)	Theoretical MW (kDa)	n
TDP(1-261)	500 + DTT	12.24	132.3	30.7	4.3
TDP(1-89)	500 + DTT	14.92	46.2	11.1	4.2
TDP(101-261)	500 + DTT	16.70	22.3	19.6	1.1
TDP(1-261)-C39/C50S	10	15.08	43.3	30.7	1.4
	50	14.92	46.2	30.7	1.5
	250	13.89	69.5	30.7	2.3
	500	13.46	82.2	30.7	2.7
TDP(1-89)-C39/C50S	10	17.32	17.9	11.1	1.6
	50	17.03	20.1	11.1	1.8
	250	16.47	25.0	11.1	2.3
	500	16.12	28.7	11.1	2.6

Figure S2. Role of the Cys residues in the oligomeric states of the N-terminal fragments as characterized by SEC. (A) SEC profiles of TDP(1-261) with or without 5 mM DTT. The protein concentration was 500 μM in Buffer A, and the

experiment was performed using a Superdex-200 Increase 10/30 GL column equilibrated with Buffer A. (B) As in (A), TDP(1-89). (C) As in (A), TDP(101-261). (D) SEC profiles of TDP(1-261)-C39/C50S at different concentrations. The right panels show the normalized chromatograms. The experiment was carried out using a Superdex-200 Increase 10/30 GL column. (E) As in (D), TDP(1-89)-C39/C50S. (F) Data from the SEC experiments (A - E) on the apparent MWs and their stoichiometries (n). Conc., concentration.

Figure S3

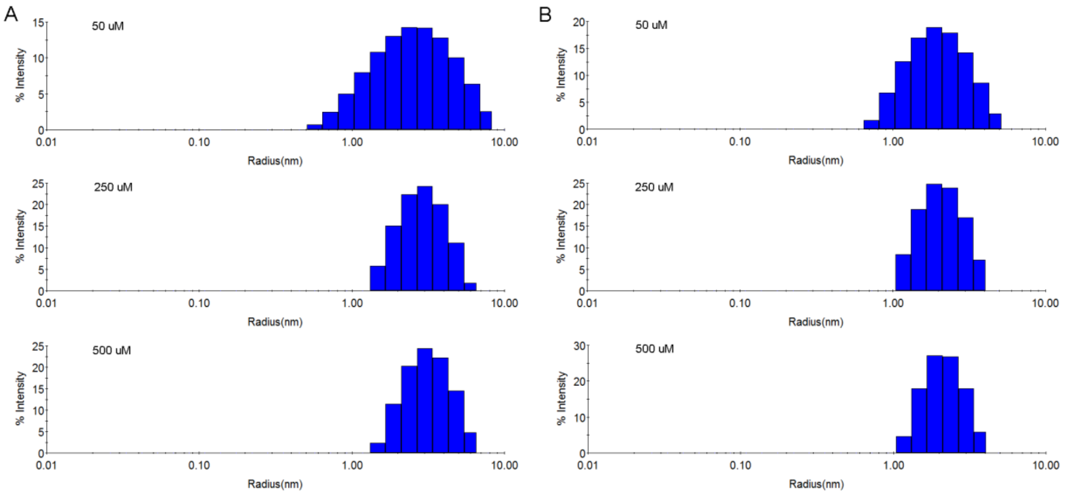

C

Protein	R_h (nm) / Apparent MW (kDa)			
	50 µM	250 µM	500 µM	Average
TDP(1-77)	2.90 / 41.3	3.01 / 44.7	3.32 / 56.0	3.08 /47.3
TDP(1-77)-C39/C50S	2.15 / 20.2	2.20 / 21.4	2.44 / 22.2	2.26 / 21.3

Figure S3. DLS analysis of the sizes and size distribution of TDP(1-77) and its Cys mutant. (A) TDP(1-77). (B) TDP(1-77)-C39/C50S. The protein was diluted with Buffer A to different concentrations, and the hydrodynamic radius (R_h) and apparent MW were calculated. (C) Data from the DLS experiments (A, B) on the hydrodynamic radii (R_h) and apparent MWs.

Figure S4

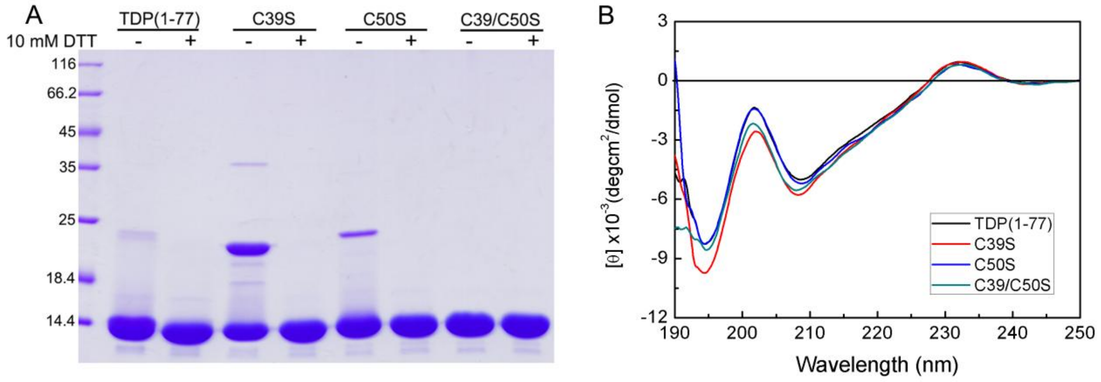

Figure S4. Effects of Cys mutations on the oligomeric states and secondary structures of TDP(1-77). (A) SDS-PAGE of TDP(1-77) and its Cys mutants with Coomassie blue staining. Each protein (500 μM in Buffer A) was treated with or without 10 mM DTT. (B) CD spectra of TDP(1-77) and its Cys mutants. The protein concentrations were 0.24 mg/mL in Buffer A.

Figure S5

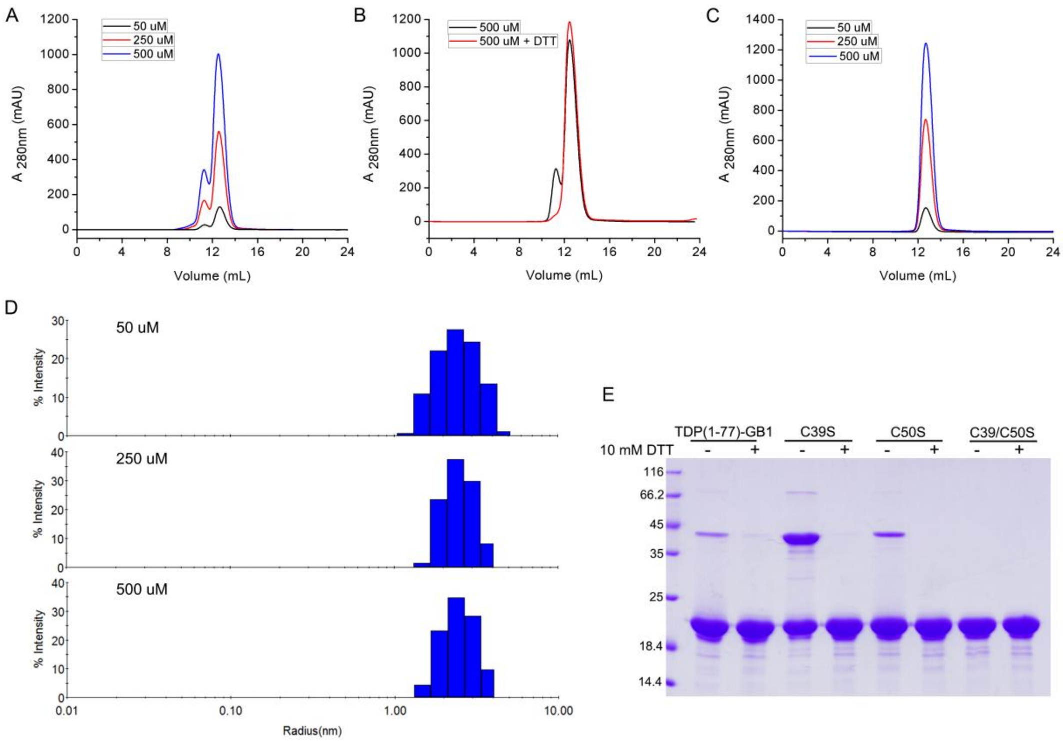

F

Conc.	50 μM	250 μM	500 μM	Average
R_h / MW (nm / kDa)	2.57 / 30.7	2.57 / 30.7	2.55 / 30.0	2.56 / 30.5

Figure S5. Characterization of the oligomeric states of GB1-fused TDP(1-77) and its Cys mutant. (A) SEC profiles of TDP(1-77)-GB1 at different concentrations. The protein was diluted with Buffer A to different concentrations and loaded onto a Superdex-75 10/30 GL column. (B) TDP(1-77)-GB1 with or without 5 mM DTT. (C) As in (A), TDP(1-77)-GB1-C39/C50S. (D) DLS analysis of TDP(1-77)-GB1-C39/C50S. (E) SDS-PAGE analysis of TDP(1-77)-GB1 and its Cys mutants. Each protein (500 μM in Buffer A) was treated with or without 10 mM DTT. (F) Data from the DLS measurements (D) on the hydrodynamic radii (R_h) and apparent MWs for TDP(1-77)-GB1-C39/C50S. Conc., concentration.

Table S1

Number of experimental restraints	TDP(1-77)-GB1-C39C50S
Total unambiguous distance restraints	829
Intra residual	447
Sequential (| i – j | = 1)	176
Medium range (2 ≤| i – j | ≤ 4)	84
Long range (| i – j | ≥ 5)	122
Hydrogen bond restraints	38
Dihedral angle restraints	
φ:	65
ψ:	64
Structure statistics	
RMSD from experimental restraints	
NOE distances（Å）	0.179±0.022
Dihedral angles (deg.)	2.706±0.161
RMSD from idealized geometry	
Bonds（Å）	0.008±0000
Angles (deg.）	0.968±0.032
Impropers (deg.）	3.026±0.184
Ramachandran analysis	
Residues in most favored regions (%)	75.1
Residues in additionally allowed regions (%)	16.0
Residues in generously allowed regions (%)	6.1
Residues in disallowed regions (%)	2.9
Average atomic RMSDs	
All residues	
Backbone atoms（Å）	0.94±0.21
Heavy atoms（Å）	1.51±0.25

Table S1. NMR experimental restraints and structural statistics for TDP(1-77)-GB1-C39C50S. The average atomic RMSD is defined from the 10 final structures.

Figure S6

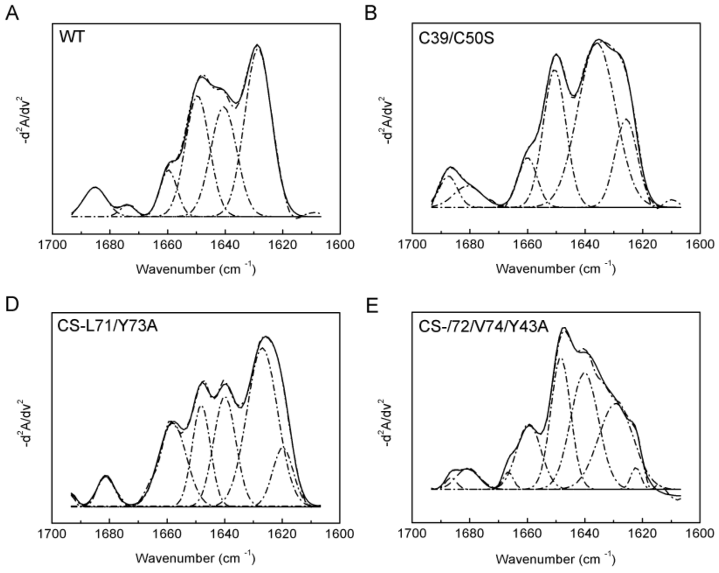

F

TDP(1-77)	β-sheet (%)	α-helix (%)	β-turn (%)	Random (%)
Wild-type	62	7	7	24
C39/C50S	61	9	7	23
CS-L71/Y73A	63	17	4	14
CS-V72/V74/Y43A	56	14	7	23
CS-L27/I60/V57A	71	8	6	14

Figure S6. FT-IR analysis of TDP(1-77) and its mutants for estimating secondary structures. (A) Curve-fitting analysis of the inverted second-derivative amide I spectrum for wild-type TDP(1-77). (B) C39/C50S. (C) C39/C50S-L27/I60/V57A. (D) C39/C50S-L71/Y73A. (E) C39/C50S-V72/V74/Y43A. (F) Secondary structure contents of TDP(1-77) and its various mutants. The secondary structure contents were estimated from the FT-IR spectra (A - E) by curve-fitting analysis.

Figure S7

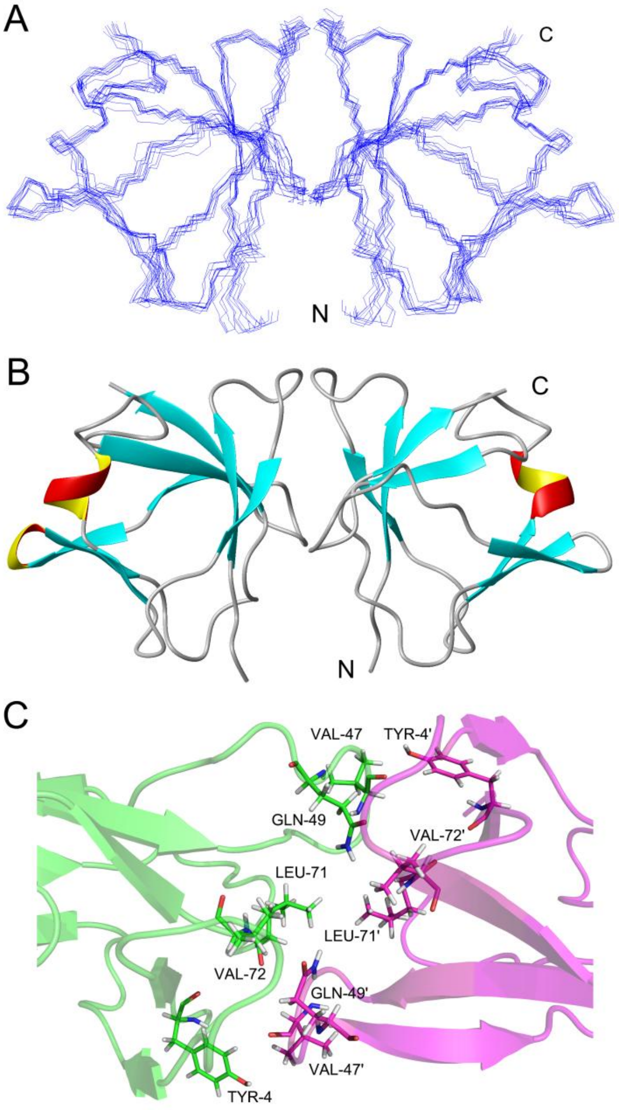

Figure S7. **Structural model of the TDP-43 NTD dimer.** The model shows the dimer interfaces centered at Leu71 and Val72 around the β7-strand. The structure was analyzed by using the HADDOCK program with the active residues from mutagenesis and SEC experiments.
